# Independent and combined effects of diethylhexyl phthalate and polychlorinated biphenyl 153 on sperm quality in the human and dog

**DOI:** 10.1038/s41598-019-39913-9

**Published:** 2019-03-04

**Authors:** Rebecca N. Sumner, Mathew Tomlinson, Jim Craigon, Gary C. W. England, Richard G. Lea

**Affiliations:** 1 School of Veterinary Medicine & Science, University of Nottingham, Sutton Bonington, LE12 5RD UK; 2 Fertility Unit, East Block B floor, Nottingham University Hospital, Nottingham, NG7 2UH UK; 3 School of Biosciences, University of Nottingham, Sutton Bonington, LE12 5RD UK; 4 Present Address: Hartpury University, Gloucester, GL19 3BE UK

## Abstract

A temporal decline in human and dog sperm quality is thought to reflect a common environmental aetiology. This may reflect direct effects of seminal chemicals on sperm function and quality. Here we report the effects of diethylhexyl phthalate (DEHP) and polychlorinated biphenyl 153 (PCB153) on DNA fragmentation and motility in human and dog sperm. Human and dog semen was collected from registered donors (n = 9) and from stud dogs (n = 11) and incubated with PCB153 and DEHP, independently and combined, at 0x, 2x, 10x and 100x dog testis concentrations. A total of 16 treatments reflected a 4 × 4 factorial experimental design. Although exposure to DEHP and/or PCB153 alone increased DNA fragmentation and decreased motility, the scale of dose-related effects varied with the presence and relative concentrations of each chemical (DEHP.PCB interaction for: DNA fragmentation; human p < 0.001, dog p < 0.001; Motility; human p < 0.001, dog p < 0.05). In both human and dog sperm, progressive motility negatively correlated with DNA fragmentation regardless of chemical presence (Human: P < 0.0001, r = −0.36; dog P < 0.0001, r = −0.29). We conclude that DEHP and PCB153, at known tissue concentrations, induce similar effects on human and dog sperm supporting the contention of the dog as a sentinel species for human exposure.

## Introduction

Over the last four decades, there has been increasing concern over declining human male reproductive health. Reduced sperm counts have been widely used as an index of male subfertility and meta-analytical studies indicate a 50% global reduction in quality from 1938 to 2011^1–3^. Sperm morphology has also been reported to decrease over a period of 17 years in France with some geographical regional variation; Aquitaine and Midi-Pyrenees having the lowest morphology combined with the lowest concentrations^4,5^. These data are indicative of an environmental aetiology and, in support of this, epidemiological studies showing increased incidences of testicular cancer and malformations at birth have been linked to regions with reduced sperm counts^6,7^.

Temporal trends in human semen quality are paralleled by a similar trend in dogs that live in the human household, where sperm motility declined by 30% over a 26 year period^8^. In this latter study, all data was generated from a single laboratory using consistent techniques and thus did not suffer from changes in methodology and quality assurance over the time span encompassed in human meta-analytical studies^9^. These observations support the hypothesis that temporal trends in semen quality, both in the human and dog, are due to shared environmental factors and that the dog may be a sentinel for human exposure to such factors. Access to a controlled breeding population of assistance dogs that are routinely sampled for sperm quality provides a cost-effective means of sperm analyses without the stigma and social complications that accompany analogous human studies. Furthermore, there is considerable potential to extend these analyses to any individual or population of dogs. For example, although not investigated in the current study, this could be achieved by semen collection from the tail of the epididymis^10^ immediately after removal of dog testes at routine surgical neutering; a procedure that is performed on hundreds of thousands of dogs worldwide each year. In addition, semen collections from live dogs is a procedure that is tolerated by a majority of breeds^11^ unaccustomed to routine fertility monitoring and can therefore easily be carried out by a trained technician.

Declining sperm quality has been linked with the exposure to persistent anthropogenic chemicals, many of which exhibit endocrine disrupting activity^6^. Although the mechanisms underlying these putative effects are uncertain, historically, the period of fetal development has been highlighted as being particularly sensitive to chemicals with endocrine disrupting activity^12^. However, a number of studies have shown that environmental chemicals (ECs) are present in semen in a range of species, including the human, raising the possibility of a direct acute effect of chemicals on sperm^13–15^. In support of this theory, an elevated concentration of seminal bisphenol A (BPA) has been associated with infertility in men^15,16^ and elevated human seminal phthalate metabolites have been associated with reduced sperm counts^17^. In a separate study, the phthalates DEHP and di-n-butyl-phthalate (DBP) in human semen were reported to be inversely associated with motility and this was confirmed by the direct application of the same phthalates, at seminal concentrations, to sperm *in vitro*^18^. By contrast, PCB congeners 118, 126 and 153 were reported to have no negative effect on human sperm motility *in vitro*, both individually and in combination^19^. Similar findings have been reported in the dog where DEHP and PCB153, at concentrations detected in dog semen and testis, exhibited inhibitory and stimulatory effects respectively when tested on sperm motility *in vitro*^8^. In the same study, both DEHP and PCB153 were detected in a range of dry and wet dog foods indicative of a dietary source. Both DEHP and PCB153 are widely present in the environment and have been detected in tissues/fluids ranging from human breast milk to ovine liver. DEHP is a widely used plasticizer that leaches out into food and liquids and PCBs are lipophilic, and are therefore present in fatty foods^20–22^. Consequently, exposure occurs largely through the diet and these chemicals are deemed as risk factors for reproductive function^23–25^. In support of this, our own published study has shown that DEHP, PCB153 and other PCB congeners are present within both dry and wet dog food sources^8^ [DEHP: wet and dry food, 0.37 ± 0.10 and 0.20 ± 0.03 μg/g respectively; ∑PCBs: wet and dry food, 1.35 ± 0.5792 and 0.78 ± 0.223 μg/kg respectively; PCB153: wet and dry food, 0.39 ± 0.193 and 0.22 ± 0.11 μg/kg respectively].

Another parameter of ejaculate quality is the proportion of sperm that exhibit DNA fragmentation^26^. Environmental chemicals have been shown to induce both human and dog sperm DNA fragmentation^8,27^ and a commercial mixture of PCBs (Arochlor), administered to rats *in vivo* and added to sperm *in vitro*, also increased sperm DNA fragmentation^28^.

In total, these data suggest that environmental chemicals induce similar acute effects on human and dog sperm *in vitro*: measurement of sperm motility and DNA fragmentation are tried and tested measures of such chemical effects. Since sperm concentration would not alter during the period of *in vitro* culture and morphology is confounded by abnormality classification, partly due to swelling that may occur during culture and the generation of artefacts during processing, these parameters were not selected for testing acute chemical effects *in vitro*^29,30^. Notwithstanding, testing the effects of individual chemicals on sperm functional parameters does not represent “real-life” exposure to a mixture of chemicals, many of which exhibit synergistic, antagonistic or additive effects. Two chemicals known to be present in dog seminal plasma and testis were therefore selected and their effects tested both independently and in combination, on sperm motility and DNA fragmentation in both the human and dog.

## Results

### Chemical effects on percentage normal sperm motility in human and dog

**Figure 1:**
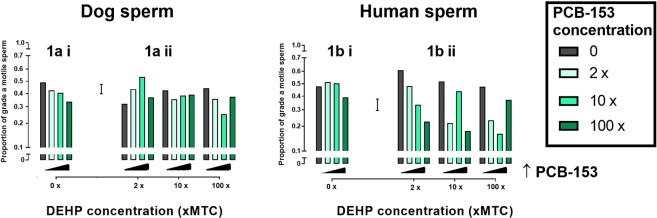
Effect of DEHP and PCB153 on dog and human sperm motility. Chemicals tested individually [**ai**,**bi**: PCB only; **aii**,**bii**: grey bars: DEHP only] and in combination [**aii**,**bii**]. Graphs display fixed concentrations of DEHP with increasing concentrations of PCB153 in dog [**ai**,**aii**: p <  0.01] and human [**bi**,**bii**: p <  0.001] sperm. Grade a motility: >25 μm/s Error bar = 1 Standard Error of Difference. MTC = Mean testis concentration.

The analyses of both the human and the dog sperm motility data found the interaction terms between PCB and DEHP to be significant (human p < 0.001; dog p < 0.05). This indicates that the dose response to either chemical was not independent of the level of the other chemical present. Figure 1 illustrates the effects of PCB153 and DEHP, individually and combined, on dog and human sperm motility. In dog sperm, PCB153 in the absence of DEHP induced a dose-dependent inhibitory effect on sperm motility (Fig. 1ai). In the presence of DEHP at 2x and 10x mean testis concentration (MTC), no dose dependent inhibitory effect of PCB153 was observed. However, reduced motility was observed in the presence of 100x DEHP co-incubated with 2x and 10x PCB153 (Fig. 1aii).

In contrast to the dog, neither PCB153 nor DEHP, in the absence of the other chemical, influenced human sperm motility (Fig. 1bi,bii). However, in the presence of 2x DEHP, a dose dependent inhibitory effect in response to PCB153 was observed (Fig. 1bii). The inhibitory effect of PCB153 was broadly maintained in the presence of 10x and 100x DEHP with the exception of 10x PCB153/10x DEHP and 100x PCB153/100x DEHP, where no inhibition was observed (Fig. 1bii).

### Chemical effects on sperm DNA integrity in human and dog

**Figure 2:**
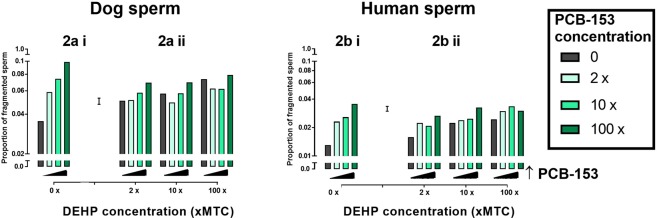
Effect of DEHP and PCB153 on dog and human sperm DNA fragmentation. Chemicals tested individually [**ai**,**bi**: PCB only; **aii**,**bii**: grey bars: DEHP only] and in combination [**aii**,**bii**]. Graphs display fixed concentrations of DEHP with increasing concentrations of PCB153 in dog [**ai**,**aii**: p ≤ 0.001] and human [**bi**,**bii**: p ≤ 0.001] sperm. Error bar = 1 Standard Error of difference between means. MTC = Mean testis concentration.

For both the human and dog DNA fragmentation data, significant (P < 0.001) PCB.DEHP interaction terms were found. Again, this indicates that the response of DNA integrity to one chemical is not independent of the presence or level of the other chemical. Figure 2 illustrates the effects of PCB153 and DEHP, individually and combined, on both dog and human sperm DNA fragmentation using the sperm chromatin dispersion assay. In the dog, PCB153 in the absence of DEHP induced a dose-dependent increase in sperm DNA fragmentation (Fig. 2ai). A similar dose-dependent increase in DNA fragmentation was observed in response to DEHP in the absence of PCB153 (Fig. 2aii). When PCB153 and DEHP were tested in combination, DNA fragmentation was still increased at higher concentrations of PCB153 but the dose-dependent response was blunted (Fig. 2aii). The response of human sperm to PCB153 and DEHP independently and combined, paralleled that observed in the dog. Figure 2b illustrates that human sperm incubated with PCB153 or DEHP in the absence of the other chemical, exhibited a dose-dependent increase in DNA fragmentation (Fig. 2bi,bii). In addition, DNA fragmentation was increased in response to both chemicals tested in combination although additive effects were not apparent.

### Sperm DNA fragmentation and motility correlations

**Figure 3:**
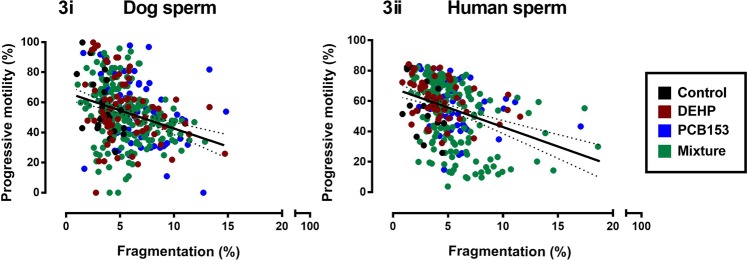
Correlation between progressive motility and DNA fragmentation in both dog and human sperm. Values from 32 sperm assessments (16 treatments, two time points). Each point represents a different sperm culture equating to a total n = 352 [dog] and n = 288 [human]. Colours denote culture media constituents to demonstrate spread: Control (black), DEHP (red), PCB153 (blue) and mixture (green). Dog (**i**, n = 352): Control; p < 0.05, r = −0.527, n = 22; DEHP; p < 0.05, r = −0.2862, n = 66; PCB153; p < 0.01, r = −0.3276, n = 66; Mixture; p < 0.0001, r = −0.2826, n = 198; vs Human (**ii**, n = 288): Control; p > 0.05, r = −0.4374, n = 18; DEHP; p < 0.05, r = −0.3118, n = 54; PCB153; p < 0.05, r = −0.2994, n = 54; Mixture; p < 0.0001, r = −0.4037, n = 162]. Confidence bands (95%) plotted for visual purposes only. Progressive motility based on WHO pre-2010 where sperm swimming grades a and b are combined: ≥5 µm/s.

Figure 3 illustrates the relationship between progressive motility and DNA fragmentation in the presence and absence of each chemical independently and combined. Despite chemical effects on sperm motility (Fig. 1) and DNA fragmentation (Fig. 2), the relationship between these two parameters remained the same regardless of the nature of the chemical exposure. All correlations were significant except the human control samples [Dog (Fig. 3i, n = 352): Control; p < 0.05, r = −0.527, n = 22; DEHP; p < 0.05, r = −0.2862, n = 66; PCB-153; p < 0.01, r = −0.3276, n = 66; Mixture; p < 0.0001, r = −0.2826, n = 198; vs Human (Fig. 3ii, n = 288): Control; p > 0.05, r = −0.4374, n = 18; DEHP; p < 0.05, r = −0.3118, n = 54; PCB-153; p < 0.05, r = −0.2994, n = 54; Mixture; p < 0.0001, r = −0.4037, n = 162].

## Discussion

Data presented in this paper are significant because we conclusively demonstrate that a selected phthalate (DEHP) and PCB congener (PCB153), at concentrations relevant to environmental exposure, reduce dog sperm motility and increase DNA fragmentation *in vitro*. In the human, our data showing reduced motility and increased DNA fragmentation are indicative of similar sensitivities to these chemicals in both species. Furthermore, when the chemicals were combined and co-incubated with dog or human sperm, the overall impact on motility or DNA fragmentation was dependent on the concentration ratio. This is the first study to select two environmental chemicals and to test them at four concentrations in all possible combinations representative of those found in the male reproductive tract and fluids. In addition, a negative correlation between motility and DNA fragmentation, that incorporates chemical variables, is described in both species.

Our data on the sensitivity of sperm to short term chemical exposure support similar studies using a range of environmentally relevant chemical challenges *in vitro*. For example, DEHP, DBP and mono-n-butyl phthalate (MBP), are reported to reduce sperm motility *in vitro* when added at seminal concentrations measured in infertile men^18,31^. A mixture of PCBs are also reported to reduce sperm motility in both the human and pig^32,33^ and in the human, p,p’-dichloro-diphenyl-dichloro-ethylene (p,p’-DDE) has been shown to increase Ca^2+^ uptake by sperm in a mechanism that involves the CatSper channel^34^.

To minimise the effects of pre-exposure to chemicals present in ejaculates collected for culture, the current study used a concentration range that encompassed the reported variability in seminal concentrations and reduced pre-existing chemical concentrations by sperm processing and washing prior to culture. The subsequent comparison to controls, with no further chemical added, provided a means of testing chemical effects. Despite these steps, pre-exposure differences will inevitably exist between, and within, species. For example, in the current study, when DEHP is present at twice the concentration found in testis, dog sperm appears to be less sensitive to lower PCB concentrations than human sperm. In populations of men from Greenland, Sweden, Poland and the Ukraine, increased seminal PCB153 has been consistently associated with reduced sperm motility but not concentration or morphology^35,36^. In addition, sperm DNA fragmentation was positively associated with PCB153 in European populations but not in samples from Greenlandic men: an observation that likely reflects different pre-exposures.

In the current study, our intention was not to measure and equate chemical concentrations in each individual sample with sperm motility and fragmentation, but to use a concentration range previously established in the dog as a ballpark estimate of chemical concentrations in the male reproductive tract^8^. That is, concentrations found in the dog reproductive tract and seminal plasma, used as an indicator of those likely present in the human. In support of this contention, human seminal PCB (total) and DEHP concentrations reported in populations of fertile men [total PCB: up to 5.8 ng/ml, DEHP: mean of 0.61 μg/ml]^37,38^ are comparable to those detected in the dog [PCB: 0.26–13.2 ng/ml; DEHP: 0.75–37.5 μg/ml]^8^.

Notably, elevated concentrations of both chemicals have been linked with reduced human male fertility^18^. Semen PCB concentrations have been reported to be higher in a population of ‘infertile’ men (parameters stated as <20 million/ml or <25% progressive motility and/or <30% normal morphology)^25^. Further, a research group in China reported an association between increased urinary phthalate metabolites, reduced sperm count and increased sperm DNA damage^14^. Although the mechanism was not proven, the authors suggest that this likely reflects chemical effects on testicular Sertoli and germ cells as reported in animal studies^39–42^.

Although both DEHP and PCB153 have been reported to reduce sperm motility and increase sperm DNA fragmentation individually in the human^8,18,37^, they have not been assessed in combination, at environmentally relevant concentrations, as reported here. A limited number of studies have investigated some combinations of environmental chemicals for their effect on human sperm function^32^. Real-life exposure is to a complex mixture of chemicals that are likely to exhibit synergistic or antagonistic, as well as additive effects, on sperm. Mixtures of endocrine disrupting chemicals have been shown to cooperatively increase Ca^2+^ concentrations in sperm through the activation of the principle calcium channel CatSper^43^.

In the current study, the mechanisms that underlie the concentration ratio-dependent effects of the two chemicals combined remain uncertain. PCBs and DEHP are considered to be pro-estrogenic and anti-androgenic respectively raising the possibility that a change in concentration ratio may alter the relative activation of sperm estrogen or androgen receptors^44,45^. Indeed, in a population of ‘infertile’ men (parameters stated as sperm count < 20 × 10^6^, motility < 50%, morphology < 14% normal) androgen receptor expression is reported to positively correlate with sperm motility^46^ and PCBs are reported to affect sperm concentration and motility relative to the number of CAG repeats in the androgen receptor gene^47^. This may account for PCB influences on motility even in the presence of DEHP. Estrogenic compounds have also been reported to reduce human sperm motility *in vitro* via an induction in redox activity and this mechanism has also been linked to the induction of DNA fragmentation by 2-hydroxy estradiol^48^.

The greater consistency of chemical effects on human and dog sperm DNA fragmentation compared to motility is interesting and emphasises the importance of looking at more than one sperm functional/viability parameter when assessing environmental effects. It is important to note however that despite this subtle difference, the two sperm parameters were highly correlated in both species and remained so in the presence of the chemicals independently and combined. This is an important observation since it has been reported that human male infertility is associated with increased levels of sperm DNA damage and that sperm motility defects are highest in samples with increased DNA fragmentation^49,50^. This raises the possibility that there may be a similar relationship between sperm DNA fragmentation, motility and fertility in the dog.

In conclusion, we have demonstrated that the use of low dose tissue relevant concentrations of DEHP and PCB153, independently and in combination, negatively impact on sperm motility and DNA fragmentation in samples obtained from humans and dogs. Since these effects are broadly similar in both species, this raises the possibility that the environmental impact of chemicals in the dog may provide a means of investigating pollutant effects on mammalian fertility in a species in which external influences, such as diet, are better controlled than in an equivalent human study.

## Methods

### Ethical Approval

#### Human

Semen donations were obtained from anonymous HFEA registered donors (n = 9) attending the fertility unit at Nottingham University Hospitals. Donors provided informed consent for the use of samples in this research project ensuring ‘General Data Protection Regulation’ compliance. Each donor was initially screened following HFEA and British Fertility Society protocols^51^. No samples from fertility patients were used and all donors completed HFEA consent forms. Ethical approval was obtained from the School of Veterinary Medicine Ethical Review Committee [Reference 1511,150723]. The HFEA consent forms completed by donors and ethical approval documents were also approved by the Chair of the Ethical Review Committee of the Faculty of Medicine and Health Sciences, University of Nottingham. In accordance with the Royal College of Pathologists guidance on the use of pathological specimens [https://www.rcpath.org/resourceLibrary/the-retention-and-storage-of-pathological-records-and-specimens-5th-edition-.html], no further ethical approval was required.

#### Dog

Semen was collected as part of routine reproductive examination of stud dogs subject to owner consent with full GDPR compliance. Due to dog sperm being collected as part of routine reproductive health checks, the economical outlay associated with sample collection was minimised. The dogs resided in the same region of the UK and lived in the modern household with owners briefed on use of controlled diet and exercise regimes. All semen collections were performed in accordance with relevant guidelines and regulations and the collection protocol was approved by the School of Veterinary Medicine Ethical Review Committee (Refs: 208 101012, 513 120117 and 1097 140227).

### Human sperm collection and preparation

Samples were liquefied for 20 minutes at room temperature prior to semen preparation. Ejaculate underwent density gradient centrifugation using a Universal 320 R Hettich centrifuge (DJB Labcare, Newport Pagnell, UK) at 25 °C. Briefly, one millilitre of 40% isotonic density gradient was loaded onto one millilitre of 80% isotonic medium [PureSperm 40/80, Nidacon, Sweden]. Two millilitres of liquefied semen were loaded onto both isotonic mediums and reagents centrifuged at 300 × *g* for 22 minutes. On completion, the sperm rich pellet was re-suspended using 1.5 ml PureSperm wash (pH range of 7.3–8.5; osmolality: 290–300 mOsm/kg H_2_O, Nidacon, Sweden).

### Dog sperm collection

Ejaculate was collected from stud dogs (n = 11) by routine digital manipulation. The sperm rich fraction (fraction 2) was collected into sterile plastic 15 millilitre Greiner centrifuge tubes [Sigma-Aldrich, Dorset, UK]. INRA extending medium [INRA, Nouzilly, France] was added to sperm at a 2:1 ratio to aid sperm survival.

### Chemical preparation

The two chemicals selected for co-culture with sperm were diethylhexyl phthalate (DEHP) and polychlorinated biphenyl congener 153 (PCB153) and concentrations were calculated relative to those present in dog testicular tissue^8^. The rationale for this was (1) dog testis concentrations of both chemicals have been established and standardised in our previous study and are reflective of exposure of the male reproductive tract^8^ (2) dog and human semen PCB concentrations are variable but generally higher than testis and in range of the concentrations tested *in vitro*^8,25,52^ (3) although dog semen DEHP concentrations have not been determined, due to the large amount of dry material required, reported human DEHP concentrations are in range of established dog testis measurements^14,18,53^. On this basis, dog testis concentrations were used as a standardised measure of environmental exposure relevant to both species. DEHP (CAS no: 117-81-7) and PCB153 (CAS no: 35065-27-1) [Sigma-Aldrich, Dorset, U.K.] were dissolved in 100% dimethylsulphoxide (DMSO) and diluted with PBS to 4x, 20x and 200x mean testis concentration containing 0.02% DMSO. Chemical preparations were co-incubated with sperm at a 1:1 ratio with a final exposure concentration of 2x, 10x and 100x mean testis concentration. Sperm were incubated with each chemical at each concentration individually and with a mixture of the two chemicals in all 16 combination ratios (Table 1). Acute treatment effects were assessed at 10 minutes and 3 hours and control incubations carried out with 0.01% DMSO only.

**Table 1:** Chemical concentrations present in culture media. ^a^MTC = mean testis concentration.

Culture	DEHP culture concentration (µg/ml) [multiple of MTC^a^]	PCB153 culture concentration (ng/ml) [multiple of MTC^a^]
Control	0.00	0.00
DEHP	0.75 [2x]	—
	3.75 [10x]	—
	37.53 [100x]	—
PCB153	—	0.26 [2x]
	—	1.32 [10x]
	—	13.22 [100x]
Mixture	0.75 [2x]	0.26 [2x]
	0.75 [2x]	1.32 [10x]
	0.75 [2x]	13.22 [100x]
	3.75 [10x]	0.26 [2x]
	3.75 [10x]	1.32 [10x]
	3.75 [10x]	13.22 [100x]
	37.53 [100x]	0.26 [2x]
	37.53 [100x]	1.32 [10x]
	37.53 [100x]	13.22 [100x]

### Motility assessment

Sperm motility was assessed using Computer Assisted Sperm Analysis (CASA) software. Sperm were acclimatised for two minutes on a 37 °C stage prior to assessment. Five µl of sperm, irrespective of species, were pipetted into a specialised 20 µl cellvision glass slide counting chamber (Code CV1020-2CV; CellVision, the Netherlands). A minimum of 200 sperm were assessed for each treatment. For human sperm, motility was tracked by use of the diagnostic software ‘SAMi’ (Procreative Diagnostics Ltd, Staffordshire, UK) and an Olympus BH2 Microscope [KeyMed (Medical and Industrial Equipment) Ltd, Essex, UK]. Dog sperm motility was assessed using the Hobson’s CASA tracking system (Hobson’s tracking systems Ltd., Sheffield, UK) and viewed using a negative-high phase contrast objective (x20) on an Olympus BH2 microscope fitted with a camera ocular. For both species, sperm motility was assessed according to WHO 1999^54^ where grade a motility was classified as ≥ 25 µm/s and progressive motility ≥ 5 µm/s (grades a & b combined).

### DNA fragmentation

Sperm DNA fragmentation was quantified by defragmentation index (sDFI %) measured using the sperm chromatin dispersion assay^55^. Sperm were assessed based on the size of the halos, indicative of sperm that exhibited nuclei with non-fragmented DNA. Sperm with denatured or fragmented DNA were identified by the absence of a halo, or a halo that was exceedingly small. Briefly, equal volumes of intact, unfixed sperm and 1% agarose solution were combined. Fifteen microliter aliquots of sperm-agarose suspensions were pipetted onto agarose pre-coated poly-L-lysine slides (CAS: P4981; ThermoFisher Scientific Ltd. UK), covered with a 22 mm × 22 mm cover-slip, and placed in a 4 °C environment for five minutes to fix the sample. After an acid treatment (0.08 M HCl) of seven minutes, sperm were incubated in a lysing reagent (0.8 M DTT, 0.4 M Tris, 2 M NaCl, 1% triton-X) for 20 minutes. Slides were then submerged in dH_2_O for a period of five minutes followed by dehydration through a series of ethanol solutions (70%, 90% and 100% respectively). Visualisation was obtained using Diff-Quick staining reagents (eosinophilic and basophilic stains; CAS 9990700, ThermoFisher Scientific Ltd, Paisley, UK) and analysis undertaken using oil immersion, at 1000x magnification [Leica DM 5000 B microscope; Leica Microsystems, Milton Keynes, UK]. A minimum of 200 sperm were assessed for each treatment. Positive controls were incubated with 300 µm H_2_O_2_ before denaturation and negative controls by omission of the denaturation step. To determine the defragmentation index, the number of fragmented sperm was divided by the total number of sperm counted. This provided a value for the proportion of sperm that were fragmented. This proportion was then plotted on the logit scale.

### Experimental design and Statistical analysis

For both the human and dog experiments, sperm samples were treated with PCB153 at one of four concentrations in combination with DEHP, also at one of four concentrations. The concentrations used for both chemicals corresponded to 0x, 2x, 10x and 100x the baseline concentration measured from dog testes. Thus there were 16 possible combinations of PCB153 and DEHP. Each combination was randomly allocated to one of 16 separate sub-samples of sperm, from each replicate donor.

Statistical analysis was undertaken using GenStat 17th edition (VSN International Ltd, Hempstead, UK). The proportions out of known numbers of sperm that had a particular characteristic were analysed as grouped binary data by fitting a generalised linear mixed model with a logit link function, assuming a binomial error distribution. The fixed effects included in the statistical model were PCB, DEHP and the PCB.DEHP interaction term. Another fixed effect, TIME, and its interaction terms were included when two repeated samples from the same tube were measured three hours apart. The random effects were donor and culture-tube within donor.

The statistical significance of a fixed effect was tested using an F-Ratio test. The analysis output provided predicted mean proportions and standard errors of difference between means which were graphically represented on a logit scale. Logit values were converted back into proportions and graphically plotted [GraphPad Prism 7.0, GraphPad Ltd, California, USA]. A single error bar on each figure represents the standard error of the difference between means. Where correlations were investigated, data was assumed to be non-normally distributed and a Spearman’s rank correlation analysis was undertaken to provide correlation coefficients between motility and DNA fragmentation.

## Data Availability

The datasets generated and analysed during the current study are available from the corresponding author on reasonable request.
